# Catching a Ball at the Right Time and Place: Individual Factors Matter

**DOI:** 10.1371/journal.pone.0031770

**Published:** 2012-02-22

**Authors:** Benedetta Cesqui, Andrea d'Avella, Alessandro Portone, Francesco Lacquaniti

**Affiliations:** 1 Laboratory of Neuromotor Physiology, IRCCS Santa Lucia Foundation, Rome, Italy; 2 Department of Systems Medicine, Neuroscience Section, University of Rome, “Tor Vergata”, Rome, Italy; 3 Center of Space Biomedicine, University of Rome, “Tor Vergata”, Rome, Italy; The University of Western Ontario, Canada

## Abstract

Intercepting a moving object requires accurate spatio-temporal control. Several studies have investigated how the CNS copes with such a challenging task, focusing on the nature of the information used to extract target motion parameters and on the identification of general control strategies. In the present study we provide evidence that the right time and place of the collision is not univocally specified by the CNS for a given target motion; instead, different but equally successful solutions can be adopted by different subjects when task constraints are loose. We characterized arm kinematics of fourteen subjects and performed a detailed analysis on a subset of six subjects who showed comparable success rates when asked to catch a flying ball in three dimensional space. Balls were projected by an actuated launching apparatus in order to obtain different arrival flight time and height conditions. Inter-individual variability was observed in several kinematic parameters, such as wrist trajectory, wrist velocity profile, timing and spatial distribution of the impact point, upper limb posture, trunk motion, and submovement decomposition. Individual idiosyncratic behaviors were consistent across different ball flight time conditions and across two experimental sessions carried out at one year distance. These results highlight the importance of a systematic characterization of individual factors in the study of interceptive tasks.

## Introduction

Interceptive actions require accurate spatio-temporal visuo-motor control of the effector. In fact, the problem of catching a flying ball is often epitomized as “getting the hand to the right place at the right time”. But what is “the right place at the right time”? In line of principle, the trajectory of a moving target could be intercepted by the moving hand at an infinite number of different spatial positions along the target trajectory, and at any time within a given temporal window. Moreover, each spatial position could be reached by means of infinitely many different hand trajectories, joint motions, and muscle activation patterns.

How the CNS copes with such redundancy is a central question in motor control, not only in the study of interception. One possibility is to reduce redundancy by constraining the available degrees of freedom. For example, when pointing to static targets, end point motions exhibit speed-independent trajectories and bell-shaped speed profiles, and systematic relations exist between shoulder and elbow joint motions [1], [2], [3], [4]. Another possibility is to select the solution, out of the many available for a given task, which minimizes a specific cost function. For example, when pointing to a long bar [5] or hitting a moving target with different velocities [6], end point trajectories are well predicted by minimizing energy, smoothness, and accuracy costs. In this context, flexible and equivalent motor behaviors may be obtained by controlling only those combinations of degrees of freedom which are relevant for successful performance [7], thus leaving most variability due to noise in task-irrelevant combinations [8].

When catching a flying ball or, more generally, when intercepting a moving object along its trajectory, redundancy in the spatial position and in the timing of interception may be reduced or exploited, depending on the specific task constraints and control strategy. For instance, the place and time of interception could be predicted before initiating the catching movement [9], [10], or the hand could move toward the target trajectory continuously guided by visual information [10], [11]. In many conditions, spatio-temporal redundancy allows for scaling movement duration and velocity [12], [13], [14], [15] or changing the interception point [16], [17] according to target speed. Adjustments of the spatio-temporal characteristics of the effector trajectory may be the result of a tradeoff between spatial accuracy, decreasing with effector speed, and temporal accuracy, increasing with effector speed [6], [13] as well as a tradeoff between variability due to sensory noise and variability due to motor noise [18], [19].

Variability in redundant tasks might arise not only from adjustments to specific constraints and because of noise, but also from differences in the control strategies employed by different individuals. For instance, one could expect that due to the large differences across individuals in sensitivity to different types of cues, such as an 80∶1(!) range in the relative sensitivity to retinal dilatation rate and binocular disparity [20], both motion planning and execution would be influenced by sensory-motor noise in a highly subject-specific manner [21], [22]. However, to our knowledge, systematic investigations of individual factors in interceptive actions are still limited. Inter-individual variability in interceptive tasks has been characterized in sport science, often in relation to the level of expertise [23], but it has been mostly overlooked in neuroscience studies. While individual differences in interception performance have been noticed [19], [24], [25], [26], [27], these have mainly been based on anecdotic observations. Indeed, when analyzing ball catching strategies, data are frequently averaged across subjects, because the emphasis is on identifying common rather than idiosyncratic features. In recent studies with visually simulated approaching balls, large individual differences in catching strategies with both constrained and unconstrained hand movements have been reported [19], [28]. However, observations with virtual targets must be confirmed in a study of unconstrained catching of real balls.

Here we investigated inter-individual variability in an unconstrained catching task. To this aim, upper limb kinematics was examined in subjects performing a one-handed catching task in which flying balls were projected with different spatial and temporal characteristics. Only subjects showing comparable success rates in interception were included in the main report. Both commonalities and differences across subjects were characterized. One of the problems when dealing with such a challenging experimental set-up is the systematic and controlled reproduction of the desired experimental conditions in the presence of air drag. To this end, we designed a launching system which was calibrated to project flying balls in space with different initial spatial and temporal characteristics. We previously showed that this system controls the desired ball flight time and arrival height with an accuracy and precision better than 96% [29].

## Methods

### Subjects

In the main report, we will detail the results obtained from six right handed subjects (5 males and 1 female, labeled S_1_ to S_6_), between 22 and 32 year old (27±3, mean ± st. dev.), selected from a group of 14 subjects (9 males and 5 females), between 22 and 47 year old (30±6, mean ± st. dev.). All 14 subjects performed a one-handed catching experiment in one or two experimental sessions carried out at about one year distance. Two exclusion criteria were used to screen volunteers as we planned to compare kinematic features independently of both performance level, and different body heights and arm lengths. First, we included only participants with good performance, that is, those who showed a standard score level as assessed in terms of a consistent number of caught trials over the total of launches (see below for a definition of “misses” and “catches”). Second, because we analyzed only caught trials which further respected a ball arrival height criterion (see Data Analysis section for details), participants who did not show at least 2 caught trials for each experimental condition were not included in the selected group of subjects. S_1_ participated only in a first experimental session (Experiment A), S_4_ only to a second session (Experiment B), and S_2_, S_3_, S_5_, and S_6_ subjects participated in both sessions. Two subjects, S_5_ and one subject from the excluded group, are respectively the first and the second authors of the manuscript. Summary results from the subjects not included in the main report (S_7_, S_8_, S_9_, S_10_, S_11_, S_12_, S_13_, S_14_) will be provided at the end of the Results section.

Participants had normal or corrected to normal vision, were informed about the procedures and the aims of the study, which was approved by the Ethical Review Board of the Santa Lucia Foundation, and gave their written informed consent to participate in the experiments.

### Task and Apparatus

**Figure 1:**
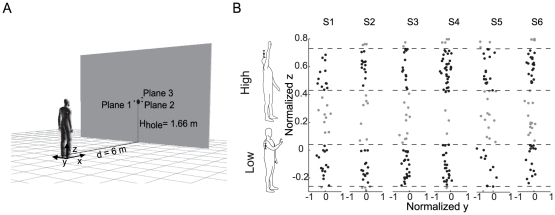
Experimental apparatus and trial selection. (A) Subjects were standing at a distance of 6 m in front of a screen with a hole through which balls were projected by a launching apparatus positioned behind the screen. (B) Ball arrival position distribution on the frontal plane for the all selected subjects and trial selection according to a normalized arrival height criterion. Scatter plots of the y-z coordinates (*frontal plane*) normalized with respect to shoulder height and arm length of all caught balls at the x coordinate of the shoulder at launch time. Trials selected for the analysis (*black dots*) are only those inside the z coordinate ranges delimited by the dashed lines. Gray dots represent trials with a normalized arrival ball height outside the height ranges.

Participants were asked to stand in front of a large screen (4×3 m, width×height), placed at a distance of 6 m from their shoulder, with their arms beside their body, and to be prepared to catch a ball launched through a hole in the screen (Figure 1A). A ball launching system was designed and constructed to automatically and precisely launch a ball from a fixed location with several initial velocities to obtain different flight conditions specified in terms of flight time (T) and height of the ball (Z) at arrival to the vertical plane at 6 m from the screen. The system has been described in details in a previous report [29]. Briefly, a commercial projection machine, conventionally used to train cricket athletes (Bola Professional Cricket Bowling Machine, Stuart and Williams, Bristol, UK), was mounted on a automated structure which allowed for vertical translation and adjustment of its elevation angle. The large screen hid the launching apparatus from the subject's view and prevented visual anticipation. The circular hole in the screen had a diameter of 14 cm and a center at an height of 1.66 m from the ground (Figure 1A). A photo-sensor (E3T-S112, Omron) mounted on the edge of the hole detected the instant at which the ball passed through the screen (launch time).

The spatial position of markers placed on the subject's head, trunk, arm and the spatial position of ball throughout its entire flight were tracked at 100 Hz using a motion capture system (9-camera Vicon-612 system, Vicon, Oxford, UK). A very large tracking volume (6×3×3 m^3^) was required for capturing the motion of both the ball and the subject upper limb. The markers reconstruction residual, averaged over the 9 cameras, obtained in such volume with the Vicon calibration procedure ranged across subjects between 0.93 and 1.01 mm (mean 0.96 mm). Retro-reflective markers were attached to the skin overlying the following landmarks: cervical vertebrae (C7); clavicle (CL); sternum (SRN); right acromion (RSHO); right epicondylus lateralis (RELB); RFRA right forearm. The middle point of a stick (length 21 cm) with two markers at the extremities (RWRU, RWRR) was taped in correspondence to the mid-point between the ulnar styloid and radial styloid. The wrist position (RW) was then computed averaging the position of the RWRU and RWRR.

Markers coordinates were referred to a right handed calibration frame placed on the floor at 6 m distance from the launch plane and oriented with the x axis horizontal and pointing from the subjects hand to the launch location and with the z axis vertical and pointing upward (Figure 1A). For safety, lightweight expanded polyurethane balls were used (weight 20 g, diameter 7 cm). The balls were covered with retro-reflective tape (Scotchlite, 3 M) to make them visible to the tracking system. The coordinates of the centre of the hole and the orientation of the screen was also estimated by means of three markers (Plane1, Plane2, Plane3 of Figure 1) placed on the screen. An additional consumer-grade PAL mini-DV video camera (MD160, Canon, 50 Hz field acquisition rate) was used to film subjects performances.

### Experimental Protocol

Six ball flight conditions obtained by the combination of three mean arrival durations (T_1_ = 0.55 s, T_2_ = 0.65 s, T_3_ = 0.75 s) and two mean ball arrival heights at d = 6 m distance from the launcher, Z_1_ (low arrival height) and Z_2_ (high arrival height), were tested. In the case of Experiment A, Z_1_ was 1.3 m and Z_2_ was 1.9 m, while in the case of Experiment B, Z_1_ and Z_2_ were adjusted according to the shoulder height of the subject (H_sh_): Z_1_ = H_sh_−0.3 m, Z_2_ = H_sh_+0.3 m.

Before each experimental session the launching apparatus was calibrated according to a procedure described in our previous report [29]. Briefly, the mapping between launch parameters and real flight characteristics at a given distance (d) from the exit hole was approximated with polynomials. The coefficient of the polynomials were fitted using the flight parameters recorded in a large number of ball trajectories obtained varying systematically the launch parameters. Finally, the launch parameters that best approximated the desired flight arrival conditions (d, T and Z) were determined taking into account that the ball launch speed could be adjusted with a resolution of 1 mph. At the end of the procedure, ball flight arrival characteristics were automatically controlled with a relative accuracy and precision larger than 98% for ball flight time and larger than 96% for ball arrival height even in the presence of large effects of air drag on the motion of the lightweight ball.

For each condition subjects performed at least 1 block of at least 10 trials each (6 blocks total). If the ball accidentally touched the ceiling of the laboratory or the edge of the exit hole on the screen, the launch was repeated. In some cases additional blocks were also performed at the end of the session. The order of the blocks was randomized across subjects. Prior to the beginning of the session subjects familiarized with the task catching a few launches with different initial conditions. Each trial started with an auditory cue to alert the subject of a new launch. After the cue the experimenter inserted the ball inside the launching machine. While the chance of visual anticipation was minimized by the screen in front of the launch machine, auditory anticipation was avoided by randomly varying the time interval, in the range of 1–2 seconds, between the cue and the insertion of the ball into the launcher.

### Data analysis

Subject's performance in the task was assessed by classifying each trial into one of three categories. A ball (and the corresponding trial) was classified as *caught* if the ball was captured by the hand, *touched* if the hand contacted but did not capture the ball, and *missed* if no contact occurred between the hand and the ball. In order to compare catching kinematics across subjects and experimental sessions in similar conditions only caught trials which also satisfied an arrival height criterion were included in the analysis. In particular, arrival height was estimated computing the intersection of the extrapolated ball trajectory with the frontal plane passing thorough the subject acromion at launch time. Caught trials were included in the analysis only if the ball arrival height, normalized with respect to subjects height and upper limb length, Z_n_, was within 30% of limb length from two reference heights whose values (Z_1n_ = −0.11, Z_2n_ = 0.58) were chosen to maximize the number of trials included in the analysis from all subjects. Limb length was computed as 1.2 times the sum of length of the arm and the forearm, where arm and forearm lengths were estimated from the positions at launch time of the markers placed on RSHO, RELB and RW. The scaling factor (1.2) was estimated measuring the position of the center of the palm in a set of three subjects (S_3_ and S_5_ from the selected group, and one subject from the excluded group). The normalized arrival height distributions for each subject is shown in Fig. 1B.

Kinematic data were digitally low-passed filtered (FIR filter; 25 Hz cutoff frequency; Matlab filtfilt function) and differentiated in order to obtain first and second order derivatives. Ball flight trajectory characteristics at a frontal plane at a specific distance (d) from the launch plane were computed fitting the ball trajectory around the position of interest with a cubic spline (Matlab csaps function) and evaluating it (Matlab fnval function) at the time of its interception with the plane. Movement was characterized by several parameters: latency time (LT), movement time (MT), impact time (IT), tau-margin reaching, first peak and first trough of the wrist tangential velocity and their time of occurrence, forearm pronosupination and elevation angles at impact, shoulder displacement, position of the ball at IT. LT was defined as the time at which wrist tangential velocity crossed a fixed threshold of 0.05 m/s. IT was computed as the instant at which the distance between the ball trajectory (spatial coordinates as a function of time) and the plane passing for the RWRU, RWRR and RFRA reached its minimum. The tau-margin reaching was defined as the time interval between the wrist peak speed and IT. Flight duration was defined as the time interval between launch and IT events. Forearm pronosupination angle was defined as the angle between the normal to the plane passing for the shoulder, the elbow and wrist markers, and the orientation of the stick applied on the wrist (0° - wrist pronated, 180° wrist supinated). Forearm elevation angle was defined as the angle between the forearm axis and the horizontal plane (0° horizontal, 90° vertical forearm). Shoulder displacement (ΔSh) during catching was assessed by estimating the difference between the shoulder position at launch and impact times.

The interception point along the ball path was quantified with an index ranging from 0 to 1. To this end, the trajectory of the ball was extrapolated beyond the interception point, up to the frontal plane (y-z) passing through the shoulder at launch time. The first possible impact point was then defined as the intersection between the ball path (projected on the sagittal plane, x-z) and a circumference of limb length radius centered on the shoulder. These two points were used to compute the interception index (I) defined as:
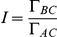
where 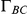 is the arc length of the ball trajectory between the frontal plane (C) and the impact point (B), and 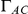 is the arc length of the ball trajectory between the frontal plane (C) and the first possible impact point (A). I = 0 means that the ball was caught in correspondence of the frontal plane, while I = 1 that the interception was in correspondence of the first reachable interception point.

Finally, we asked whether and how subjects modulated their hand movement along the main direction of motion (z axis), depending on the object motion. Thus, we also looked at the hand-ball kinematic coupling and analyzed the hand vertical velocity relative to the ball vertical velocity at the time of impact for each one of the six experimental conditions.

### Submovement decomposition

Submovement decomposition was carried out to investigate the possibility of a similar structure underlying the kinematics observed in different subjects. In particular we tested whether the observed tangential velocity profiles could be reconstructed by the same number of minimum-jerk subcomponents differently shifted in time and scaled in amplitude and duration.

**Figure 2:**
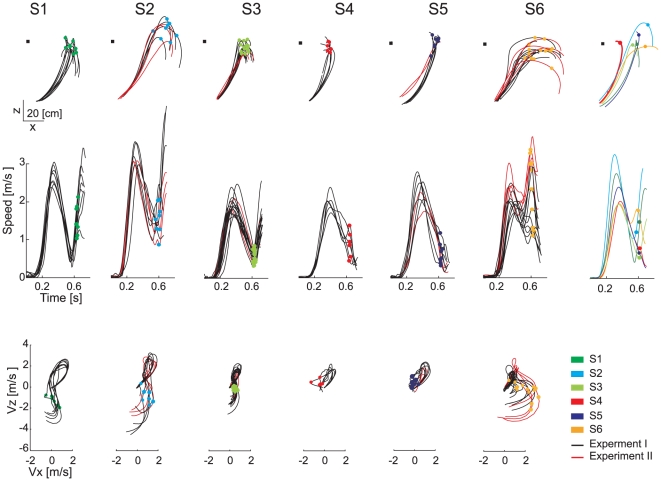
Example of wrist kinematic differences across selected subjects. Wrist trajectory (*first row*), x-z tangential velocity profiles (*second row*), and velocity components in the sagittal plane (*third row*) are shown for individual trials of each subject (columns 1–6) in the T_2_Z_1_ condition as well as averaged across trials for each subject (column 7). Black lines are relative to the trials recorded in a first experimental session. Red lines are relative to trials in the same experimental condition recorded during a second session carried out one year later. All trajectories are plotted up to 100 ms after the impact event, and translated to align the shoulder position at launch time (indicated by the black square).

For each trial, the wrist speed profile was decomposed into the smallest number of submovements that permitted to reconstruct the original profile with a R^2^ value larger than 99%. Since the wrist path was found to be highly curvilinear (see Figure 2), the use of tangential speed was thought to be more appropriate in capturing the different phases of the movement with respect to velocity components used in previous studies dealing with similar issues [27]. We assumed that each submovement had a minimum jerk speed profile, 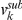, with variable amplitude (A), duration (D), and onset (t_0_):
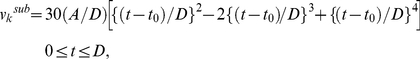
(1)hence, the entire speed profile was fitted by the combination of *N* sub-movements
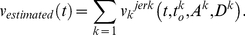
(2)


Similarly to previous studies [30], [31] we implemented an algorithm to determine, for each *N*, the amplitude, onset and duration of each submovement that minimized the error between estimated and real speed profiles. In particular, we used a scattershot optimization algorithm which pursued a local optimization starting from a number of random initial conditions (fmincon Matlab function) where the probability of finding the best submovement composition increased as the number of random initial conditions increased. For a given *N*, the best over 20 different runs of the optimization algorithm with different random initial conditions was selected. At the *i*-th run, all the *N* submovements parameters were initialized with the same A_i_ (A_M_/N, where A_M_, is the total movement amplitude) and D_i_ (MT/N) but different t_0i_, randomly sampled from a Gaussian distribution centered at equi-spaced times and with σ = D_i_/2. Furthermore we constrained the possible values of A_i_ in the interval [0.1: A_M_] and the possible values of t_0_ within the interval [(LT−minD); (MT−minD)], where minD, was the minimal submovement duration, set equal to 100 ms. In addition, we imposed an ordering to the onsets and offsets of the components (t_0k_<t_0k+1_, t_offk_<t_0k+1_, 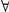
k 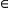
[1,(N-1)]). We then determined the smallest number of submovement *N* necessary to fit the data with the required accuracy.

Submovements were fitted in two different time windows depending on the shape of the speed profile. In particular there were two possibilities: 1) subcomponents were fitted within [Won, IT+0.1 s] in all cases where the wrist speed profile never went below a threshold of 0.05 m/s during the 100 ms interval following the impact; this countermeasure was adopted in order to be sure to correctly extract the parameters (max peak speed, duration, and onset) of the last submovement when subjects did not stop on the ball at impact; 2) subcomponents were extrapolated within [Won, IT] when subjects tended to stop on the target at impact.

### Statistical analysis

To explain the effect on catching performance of experimental condition (three ball flight times and two ball arrival heights), of practice (i.e. the trial number within each block), and of subject, the response variable Y, indicating caught (Y = 1) and non-caught (Y = 0) balls, was modeled with a Generalized Linear Mixed Model (GLMM) [32]. Similarly, all kinematic parameters analyzed for the trials selected according to the criteria described above were modeled with a Linear Mixed Model (LMM) [33], [34]. GLMM and LMM allow for testing simultaneously for both fixed effects (ball flight time, arrival height, and trial number), and random effects (subject). However, for each subject, a standard multiple linear regression analysis was also performed on all main kinematic parameters to test for effects of practice (including only the first 10 trials of each block) and performance.

Statistical analyses were performed in R software environment (R development Core team (2011). R foundation for statistical computing, Vienna. ISBN:3-900051-07-0 URL http://www.R-project.org) with the lme4 package (lme4: Linear mixed-effects model using S4 classes. R package version 0.999375-39. http://CRAN.R-project.org/package=lme4). The restricted maximum likelihood estimation (REML) was used to fit the models [32]. In the case of LMM, significance of fixed effects was assessed using a Markov Chain Monte Carlo [32]. The level of significance was set as p<0.05. To evaluate whether there were differences across subjects, we compared the Akaike Information Criterion (AIC) for the LMM, i.e. including random effects, with the AIC value computed for a linear model (LM) including only fixed effects. The AIC evaluates the quality of the fit taking into account the complexity of the model (the lower the AIC value, the better the model fitting, see [32], [33]). If the AIC_lmm_ resulted lower than the AIC_lm_ the inclusion of the subject factor was justified, providing evidence for inter-individual differences. For our purpose the AIC test is preferable to other criteria such as the Likelihood-Ratio test which has been found to be not appropriate to evaluate whether a random factor should be included in the model [35], [36].

## Results

### Performance

Ball flight time and arrival height affected the number of caught balls. Analysis based on a GLMM showed a significant fixed effect of T and Z on the trial success (response variable Y). In particular, the number of caught balls increased as T increased and Z decreased in accordance with previous studies [15] (T: β_T_ = 7.51, p<0.01; Z: β_Z_ = −1.4, p<0.01). Moreover, the number of caught trials differed across subjects (AIC_glmm_ = 426.3, AIC_glm_ = 432.08). However this difference was due to a single subject (S_1_) who performed slightly better than all others. A second analysis carried out excluding S_1_ showed that performance was not different across the remaining 5 subjects (AIC_glmm_ = 364.8, AIC_glm_ = 362.8). Furthermore, the trial number significantly affected the response variable Y (p<0.01). This effect was due to the fact that subjects failed to catch the ball in the first trial more often than in the subsequent trials of each block. However, a second analysis carried out removing the first trial of each block showed no significant effect of trial number on the response variable (p = 0.06), indicating that practice did not affect performance. Moreover, neither practice (i.e. trial number within each block) nor performance (i.e. response variable Y) affected any of the hand kinematics parameters described below, as multiple regression coefficients were not significant in all subjects.

### Wrist kinematics features

Large differences in the kinematic features of interceptive movements were observed between subjects. Examples of wrist trajectories, tangential velocity profiles, and velocity components in the sagittal plane for all individual trials of each participant in the T_2_Z_1_ condition are shown in Fig. 2. Each subject appeared to intercept the ball with a different strategy. S_1_ and S_2_ presented hook-like wrist trajectories with an initial upward component, characterized by the highest peak tangential velocity recorded across participants, followed by a second downward component. In particular, S_1_ first raised the wrist quickly up to shoulder height, positioning the hand in the “catching zone”, and then started accelerating downward, and impacted the ball close to the shoulder. S_2_ in contrast caught the ball far away from his shoulder after raising the wrist higher than the final interception point. S_3_ also presented hook-like wrist paths and accelerated the wrist downward immediately after impact but, differently from S_1_ and S_2_, caught the ball in correspondence of the highest point of the wrist path and the minimum of wrist speed. S_4_ and S_5_ moved directly toward the target approaching the interception point from below and showed a low vertical impact velocity. S_4_ caught the ball close to the shoulder, while S_5_ caught the ball further away from the body. In most cases, S_4_ and S_5_ had tangential velocity profiles with only one peak. Finally, S_6_ initially raised the wrist slower than the other subjects and then moved forward and slightly upward, and captured the ball further away from his body, often exactly at the time of the second wrist speed peak.

The particular subject-specific kinematics observed in one condition were similar within all flight time conditions with low ball arrival height. Furthermore, subject-specific kinematic features were retained over time as shown in Figure 2 for S_2_, S_3_, S_5_, and S_6_ by the red lines corresponding to the x-z wrist trajectories, tangential velocity profiles, and x-z velocity components recorded with the same T-Z flight arrival conditions during a second experimental session performed one year after the first session. Similar conclusions were also valid for high launches: wrist trajectories characteristics were consistent across flight time conditions, although differences were less evident than for low launches, as will be further described below.

### Wrist velocity time-course

**Figure 3:**
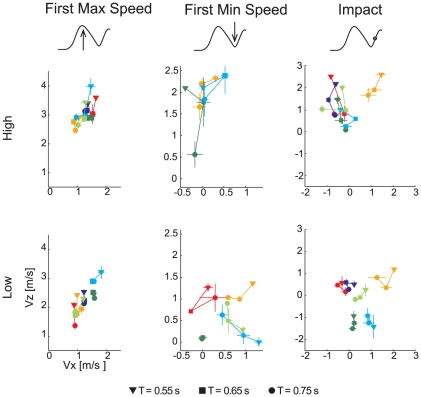
Inter-individual differences in wrist velocity at maximum speed, minimum speed, and impact. Wrist velocity components (mean ± SE; SE are reported only when number of trials per block was larger than 2) in the sagittal plane (x, anterior-posterior axis; z vertical axis) for each one of the three flight time conditions (T, indicated by different marker shapes) are illustrated separately for the two different arrival heights (*first row*: high, *second row*: low). Subject color coding is the same as in Figure 2.

Individual kinematic features were quantified by monitoring the components of the wrist velocity in the sagittal plane at three different instants: the time of the first speed peak, the time of the first speed trough, if present, and the time of impact. Inter-subject variability increased getting closer to IT, as highlighted by the progressively broader distribution of velocity components (Figure 3). However, subject-specific characteristics were present since the beginning (AIC_lmm_<AIC_lm_ for all x-z velocity components, v_x_ and v_z_, at the three time instants of interest, see Table 1). For low launches (bottom panels of Figure 3), S_1_ and S_2_ presented higher values of v_x_ and v_z_ at the time of peak wrist speed, and lower values at the time of minimum speed with respect to the other participants. S_1_ (*dark green*) presented a segmented motion, and decelerated to almost 0 velocity before the final downward displacement of the hand, as indicated by the small v_x_ and v_z_ in correspondence of the first minimum of the wrist speed. S_2_ (*light blue*) showed instead higher v_x_ both in correspondence of the peak and the trough of the speed profile, probably due to his tendency to move smoothly further toward the approaching target. S_6_ (*orange*) instead began to manifest its strategy in correspondence of the first minimum of the speed profile, when he moved at higher velocity than the rest of population. The analysis of the wrist velocity at IT showed that subjects caught the ball with very different horizontal and vertical components (third column of Figure 3). In low launches both S_1_ and S_2_ presented a large negative v_z_ due to the downward motion before the impact. Differently from S_1_, S_2_ moved toward the ball with also a higher v_x_. S_3_ (*light green*), S_4_ (*red*), and S_5_ (*dark blue*) impacted the ball with a low velocity both along the x and z axis. In particular, S_5_ showed a negative v_x_, i.e. he moved slightly backward at impact, S_3_ instead stopped on the ball, and S_4_ tended to move toward the ball with a positive v_x_. Finally, S_6_ showed very high positive v_x_ and v_z_ due to the final forward and upward motion aimed at catching the ball from below.

**Table 1:** Effect of fixed and random factors on kinematic parameters (LMM analysis).

	Intercept		T [s]			Z [m]			AIC	
KinematicParameters	β_o_	P_I_	β_T_	β_T_ 95% C.I.	P_T_	β_Z_	β_z_ 95% C.I.	P_Z_	AIC_lmm_	AIC _lm_
LT [s]	0.06	0.04	0.05	[−0.03; 0.13]	0.21	0.02	[−0.01; 0.04]	0.17	−671.30	−690.60
peak speed [ms^−1^]	2.61	***	−3.43	[−4.07; −2.8]	***	1.35	[1.77; 1.53]	***	134.50	219.31
T to peak [s]	0.08	0.018	0.20	[0.11; 0.28]	***	0.02	[−0.01; 0.04]	0.12	−674.60	−624.50
tau-margin reaching [s]	−0.22	***	0.73	[0.66; 0.79]	***	0.02	[0.01; 0.04]	0.02	−797.10	−748.31
v_x_ at peak speed [ms^−1^]	2.37	***	−1.27	[−2.31; −0.29]	0.01	−0.6	[−0.86; −0.35]	***	294.1	294.73
v_z_ at peak speed [ms^−1^]	0.3	0.62	−5.51	[−7.09; −3.91]	***	2.9	[2.44; 3.28]	***	482.6	539.19
v_x_ at minimum speed [ms^−1^]	2.44	***	−0.46	[−1.4; 0.48]	0.33	−1.13	[−1.36; −0.88]	0.00	97.50	142.09
v_z_ at minimum speed [ms^−1^]	−0.69	0.28	−3.53	[−5.24; −1.78]	***	2.39	[1.96; 2.82]	***	229.30	257.18
v_x_ at IT [ms^−1^]	2.67	***	0.04	[−1.12; 1.06]	0.97	−1.42	[−1.7; −1.12]	***	333.30	490.00
v_z_ at IT [ms^−1^]	−0.89	0.03	−5.60	[−6.54; −4.62]	***	2.80	[2.54;3.034]	***	280.10	481.14
T_C_−T_B_ †† [s]	0.065	***	0.09	[0.07; 0.11	***	−0.05	[−0.05; −0.04]	***	−1165	−987.9
interception index	0.56	***	0.68	[0.41; 0.97]	***	−0.29	[−0.37; −0.22]	***	−206.10	−86.71
wrist pronosupination angle [deg]	102.3	***	−3.83	[−65.04; 62.57]	0.92	−25.43	[−43.86; −7.85]	0.01	1784.00	1813.05
elbow elevation angle [deg]	11.79	0.05	4.25	[−10.74; 18.59]	0.61	25.36	[21.27; 29.40]	***	1247.00	1356.87
ΔSh_x_ [m]	0.11	***	0.09	[−0.1; −0.06]	0.03	−0.08	[−0.1; −0.06]	***	−675.00	−599.63
ΔSh_z_ [m]	−0.29	***	0.02	[−0.03; 0.08]	0.39	0.18	[0.16; 0.19]	***	−775.20	−697.15
Number ofSubmovements	2.93	***	1.51	[0.54; 2.53]	***	−0.83	[−1.08; −0.56]	***	285.3	341.51
v_x_ at IT (all 14 subjects) [ms^−1^]	2.76	***	−0.82	[−1.3; −0.42]	***	−1.21	[−1.32; −1.11]	***	1414	2028.61
v_z_ at IT (all 14 subjects) [ms^−1^]	0.045	0.79	−4.83	[−5.35; −4.32]	***	2.18	[2.04; 2.31]	***	1726	2370.33

Different columns report the regression coefficients (β) and p-values (P, evaluated by MCMC sampling, 10.000 simulations, see Methods and [32]) for the intercept and the fixed factors (flight time T, ball arrival height Z). The two rightmost columns report the AIC values computed including the random factor (AIC_lmm_) and without it (AIC_lm_). If AIC_lmm_>AIC_lm_ the inclusion of the random factor (i.e. subject) is justified, indicating that the particular kinematic parameter varies across subjects. Results are from the six subjects selected in the main study with the exception of the last two rows referring to all the 14 subjects enrolled in the experiment.

**Figure 4:**
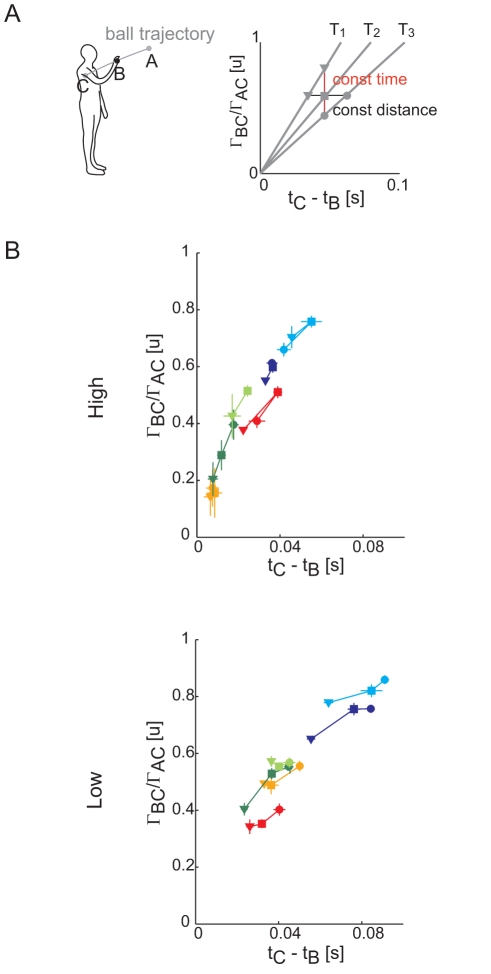
Interception point along the ball trajectory. (A) *Left panel*: schematic representation of the interception point index (computed as the ratio between the BC and AB ball trajectory arc lengths, see Methods). *Right panel*: for each flight time condition (T_1_–T_3_), subjects impact point along ball trajectory (normalized with respect to subject arm length) was uniquely determined by the movement time, hence the value of the difference between the extrapolated time of arrival of the ball at the frontal plane (t_C_) and the impact time (t_B_). However, for different flight times, subjects are free to vary t_B_ and impact the ball at the same normalized distance (vertical line labeled “const distance”) or to catch the ball closer to their shoulder (horizontal line labeled “const time”). (B) Scatter plots of the interception index vs. t_C_−t_B_ (mean ± SE across trials in the same conditions; SE are reported only when number of trials per block was larger than 2). A value of I = 0 indicates a catch at C while I = 1 indicates a catch in correspondence of the first possible interception point (A point), computed as the interception between the ball trajectory and the sphere centered at the shoulder joint of radius equal to arm length. It was not possible to determine S_3_ behavior in the T_3_Z_2_ condition because the shoulder marker detached and was missing throughout the entire block. Subject color coding as in Figures 2 and 3.

***: p_value<0.01;

†† T_c_ = time of arrival at the frontal plane passing for subject shoulder at launch time; T_B_ = impact time (see Figure 4).

In the case of high launches, inter-individual differences were less marked in the initial part of the movement, yet still evident. Indeed, all subjects caught the ball with a positive vertical velocity, did not stop, and, with the exception of subjects 2 and 6, impacted the ball with a negative v_x_. However, some of subject-specific characteristics observed in the low launches were still present: for example, S_6_ moved faster in the x direction compared to the other participants.

LMM analysis showed that the effect of flight time and ball arrival height was significant on v_z_, at all three different time instants evaluated. In particular subjects slowed their vertical movements as T increased (β_T_<0) and Z decreased (β_Z_>0). On the contrary, v_x_ did not depend on T, except at the time of peak speed, but depended on Z, as subjects significantly decreased their horizontal velocity at higher ball arrival heights (β_Z_<0).

### Impact point

Different participants caught the balls at different times and positions along the ball trajectory (Figure 4B). Comparison of LMM and LM models confirmed that both the impact index (Γ_BC_/Γ_AC_) and the relative impact time (t_C_−t_B_) differed across subjects (AIC_lmm_<AIC_lm_, see Table 1). In addition each subject showed a different spatial distribution of impact points across ball arrival time and height conditions. In this respect, given that the impact point along the ball trajectory, i.e. the interception index Γ_BC_/Γ_AC_ (see the schematic plot in the right top panel of Figure 4A), for each ball flight time is uniquely determined by the impact time, i.e. the t_C_−t_B_ value, subjects were free to intercept the balls with different flight times either at the same normalized distance from the frontal plane, i.e. the same Γ_BC_/Γ_AC_ value (vertical line labeled *const distance* in the panel), or at the same impact time but with different Γ_BC_/Γ_AC_ values (*const time*), or at different distances and times for each ball flight time.

LMM analysis showed a significant fixed effect of flight time and ball arrival height both for the impact index and the t_C_−t_B_ values (see Table 1). In accordance with previous reports [16], [17] both values increased with increasing T, i.e. subjects tended to intercept the target closer to the shoulder when facing faster balls (impact index: β_T_ = 0.68; t_C_−t_B_: β_T_ = 0.09). In contrast, both values decreased for higher launches (β_Z_ = −0.29). Such a behavior could be explained by the stricter temporal and spatial constraints present in the ball trajectories for high launches with respect to the low ones. Indeed for high launches both the temporal (t_C_−t_B_) and spatial (Γ_AC_) windows were reduced: the three T lines in the right panel of Figure 4A become closer to each other, implying a smaller length of the ball trajectory within arm reach.

In low launches, S_4_ (*red*) intercepted the target close to his body, later than the other participants, and halfway from the first possible impact point and frontal plane, with interception index ranging between 0.4 and 0.5 (Figure 4B). In contrast, S_2_ (*light blue*) and S_5_ (*dark blue*) caught the ball earlier and closer to the first possible interception point with respect to the other subjects, and showed an index ranging between 0.65 and 0.86. The rest of the subjects caught the ball at intermediate interception index ranging between 0.6 and 0.75 (see Figure 4B). [16], [17]. In high launches, S_2_, S_3_, S_4_ and S_5_ maintained impact indexes values similar to those observed in low launches. Only S_1_ (*dark green*) and S_6_ (*orange*) caught the ball closer to their body than in low launches with an index ranging between 0.14 and 0.40. Overall subjects appeared to use a strategy in between the *const time* and *cont distance* strategies. However each subject preferred a specific “catch zone” along the ball trajectory.

### Upper limb posture and trunk motion

LMM analysis showed that there were inter-individual differences on the limb posture at impact (AIC_lmm_<AIC_lm_ both for wrist pronosupination and elbow elevation parameters, see Table 1). Moreover, there was an effect of ball arrival height (elbow elevation increased, β_z_ = 25.36, and wrist pronosupination decreased, β_z_ = −25.36, with Z) and no significant effect of flight time.

**Figure 5:**
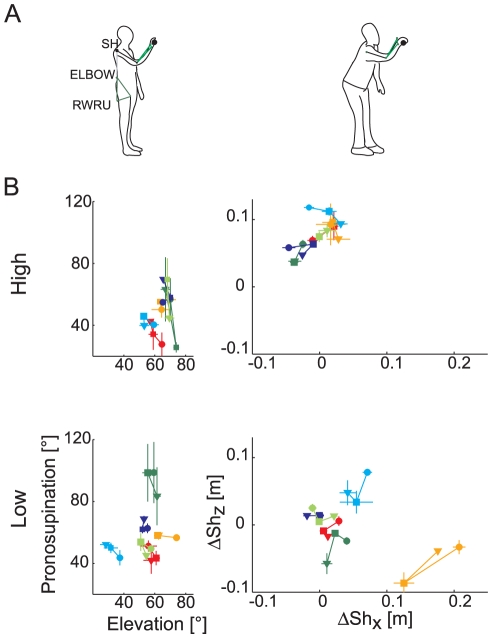
Wrist posture and trunk displacement at impact. (A) Examples of different body and arm postures at impact in two subjects (S_5_ *left*, S_6_ *right*). (B) *Left panel:* scatter plots of the forearm pronosupination angle vs. forearm elevation angle (mean ± SE across trials of the same condition; SE are reported only when number of trials per block was larger than 2). *Right panel*: shoulder displacement in the sagittal (x-z) plane between launch and impact times. It was not possible to determine Subject 3 behavior in the T_3_Z_2_ condition since the shoulder marker detached and was missing throughout the entire block. Subject color coding as in Figures 2 and 3.

In low launches, S_1_ presented a pronosupination of almost 90° and an elevation angle ranging between 50° and 60°, which corresponded to an arm posture with the palm plane parallel to the sagittal plane (Figure 5, *left panel*). S_2_ tended to catch the ball from above, with the arm parallel to the horizontal plane and the forearm pronated. S_3_, S_4_, and S_5_ caught the ball with a forearm elevation angle ranging from 40° to 60° and a pronosupination angle ranging from 40° to 70°. This arm posture corresponded to a configuration in which the normal to the palm plane was almost parallel to the x-axis and the arm was not fully extended (more evident in S_5_ than in S_3_ and S_4_), as illustrated by the line drawing in the left panel of Figure 5A. Finally, S_6_ brought the forearm in a more vertical orientation with respect to the other participants and with the palm parallel to the frontal plane (see the line drawing in Figure 5A, *right*).

As for the other kinematic features described above, differences across subjects in arm posture were less marked in high launches. Almost all subjects caught the ball with a similar wrist-limb postural configuration, i.e. with the arm fully extended along the vertical axis and the palm oriented parallel to the frontal plane. The higher mean value and standard error showed by S_3_ in condition T_3_Z_2_, was related to the presence of a few trials with a slightly lateral component (along the y axis), which required to abduct the shoulder modifying the arm-wrist configuration of the catch.

There were differences among subjects also with respect to the exploitation of the trunk (Figure 5, *right panels*) as indicated by the comparison between with (LMM) and without (LM) random effects models (AIC _lmm_<AIC_lm_, Table 1). In low launches, S_1_ remained in the same position with respect to the x-axis, but tended to stoop a little. On the contrary, S_2_ slightly advanced the shoulder along the positive×direction, in line with his preference to move toward the approaching target, and raised the shoulder. S_6_ showed a considerable forward and downward motion of the shoulder during the catch (line drawing on the right panel of Fig. 5A). In contrast, S_3_, S_4_ and S_5_ did not involve the trunk in the interception motion. In the high launches a positive vertical displacement of the shoulder was required in all subjects; a slightly backward motion (negative displacement along the x axis) was observed in S_1_, S_3_, S_4_ and S_5_.

### Ball-hand coupling

While we found a striking inter-individual variability in the kinematic features of interceptive movements we also found dependences of movement kinematics on flight conditions similar to those reported in previous studies [15], [16], [17], [37], [38] (see Table 1). LT was not significantly affected by ball arrival time and height (p_T_ = 0.21; pz = 0.17) and did not vary across subjects (AIC_lmm_>AIC_lm_). On average the movement was initiated 0.12±0.04 s after the launch. On the contrary, fixed effects of flight time (T) and ball arrival height (Z) on wrist peak speed were significant, showing that subjects increased wrist peak speed with Z (βz = 1.35) and decreased it with T (β_T_ = −3.43). Time to peak duration increased with flight time, (β_T_ = 0.2) while main effect of ball arrival height was not significant. Finally, tau-margin reaching increased with increasing of flight time and ball height (β_T_ = 0.73, β_Z_ = 0.73). In line with previous results there were differences across subjects as shown by the AIC comparison (AIC_lmm_<AIC _lm_).

In sum, while participants showed a similar modulation of kinematic features of their movement as a function of temporal and spatial constraints of the task, we found substantial differences across subjects in their interceptive actions.

### Submovement decomposition analysis

An analysis of the submovement composition of the catching movements was undertaken to gain additional insights on the subject-specific strategies. Previous studies have suggested that the control of discrete movement components could be guided by the information on target motion characteristics [27], [39]. We found that subject-specific wrist kinematics corresponds to specific movement structures that differed in the number and parameters (onset, amplitude, and duration) of the components.

**Figure 6:**
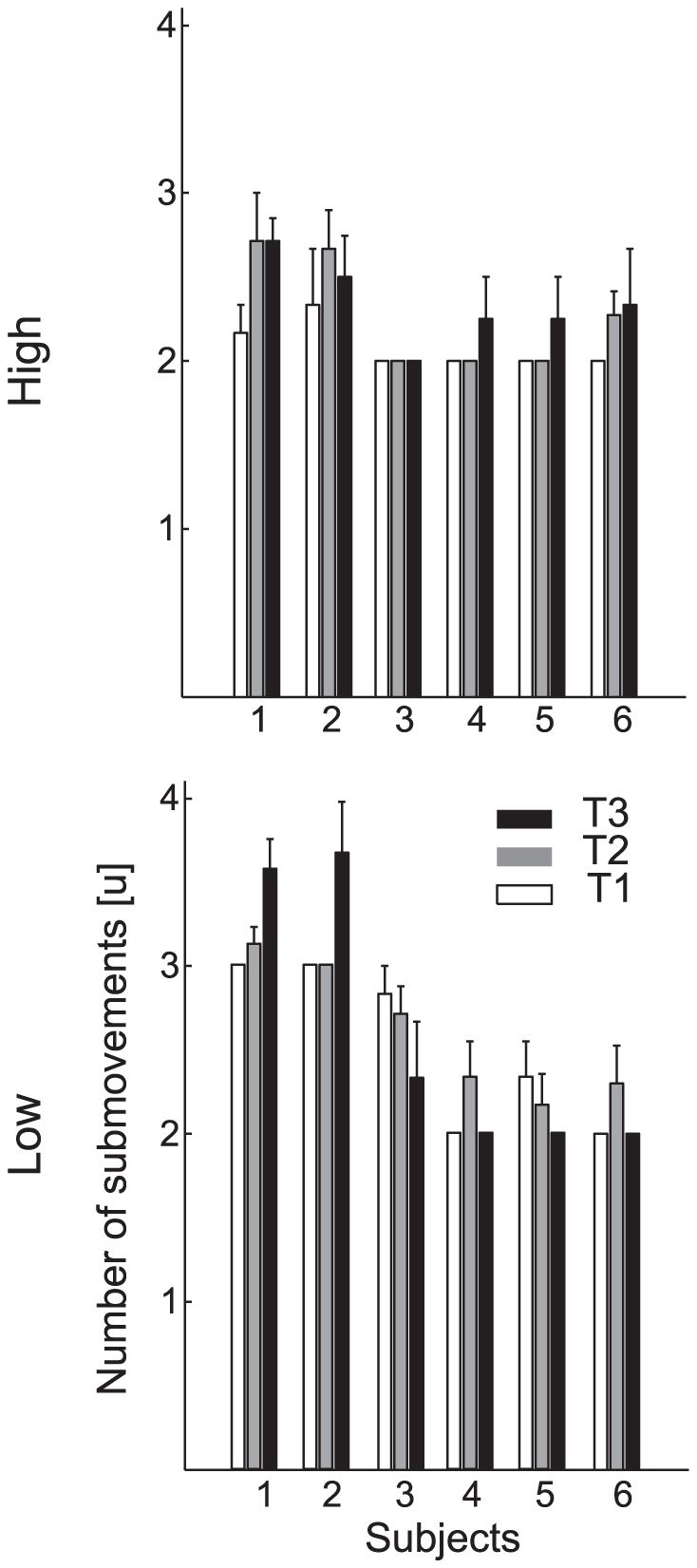
Frequency of the submovement components. Mean and SD across trials of the number of submovement components extracted by the algorithm for the two ball arrival heights (different *rows*) and three flight times (different *shading*) for each subject. Different subjects showed different submovement structures characterized by different mean numbers of submovements.

**Figure 7:**
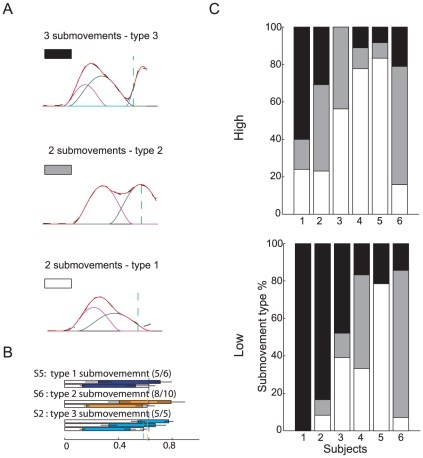
Classification of movements according to submovement composition. (A) Examples of the 3 types of submovement decomposition characterizing all observed wrist velocity profiles. (B) Submovement duration distribution in the T_2_Z_1_ experimental condition, for 3 of the 6 selected participants, representative of the first three movement types. Each submovement is reported in a different line. The colored bars represent the mean duration and the horizontal lines the SD of the onset and of the offset of each submovement. Subjects presented a robust behavior across trials of the same block, as shown by stable segments duration distribution. (C) Frequency distribution of submovement types (expressed in percentage) for each ball arrival height and for each subject; different hand speed profile decomposition structures were sometimes observed when catching lower or higher targets, as in the case of subjects 2 and 3.

The mean number of submovements per trial (Figure 6) varied across subjects (AIC_lmm_<AIC_lm_, see Table 1), ball arrival heights and flight time conditions. Overall subjects increased the number of submovements for slower balls (β_T_ = 1.51) and decreased it for higher launches (β_Z_ = −0.83) We classified each movement according to the results of its submovement decomposition into 3 groups (Figure 7A). In particular when the speed profile presented only one peak or the total number of submovement (*N*) was 2 and the peak of the second submovement occurred before the speed trough, the movement was classified as type 1. In this type of movements the second component showed a longer duration and a smaller amplitude than the first one, and it was responsible for a gently decelerated movement toward the target. When instead *N* was 2 and the peak of the second submovement occurred after the speed trough, the movement was classified as type 2. In this group a first submovement was generated to bring the hand closer to the “catch zone”, while a second submovement was triggered and opportunely timed to bring the hand on the target in correspondence of its peak. Finally when the total number of submovement was 3 or 4 movements were classified as type 3. In type 3 movements, the first two components were responsible for bringing the hand roughly in the interception zone, with an initial submovement raising the hand, followed by a higher amplitude and longer second submovement during the deceleration phase, while the third and fourth components, with a smaller duration and higher amplitude then the previous two, brought the hand on the ball. Subjects whose wrist tangential velocity profile had most often a single peak (S_4_ and S_5_) showed a prevalence of movements of type 1 (Figure 7C). Subjects who approached the ball from below and showed a positive vertical velocity at impact, had speed profiles most often classified as type 2. This often occurred for speed profiles with two peaks and with a high speed values at the trough between them (see Figure 2 and 3, S_6_ and S_4_ in low launches). Finally, subjects approaching the ball with a hook-like wrist trajectory, showed a higher percentage of trials with movements of type 3. For high launches those differences were less evident due to the fact that in most of cases movements were of type 1 and type 2.

### Comparison with the group of excluded subjects

**Figure 8:**
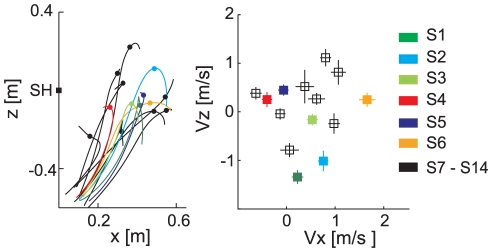
Example of wrist kinematic features of excluded subjects. *Left panel:* wrist trajectory averaged across all caught and intercepted trials in the T_2_Z_1_ condition are shown for all 14 subjects enrolled in the study; all trajectories are plotted up to 100 ms after the impact event, and translated to align the shoulder position at launch time (indicated by the black square). *Right panel*: velocity components in the sagittal plane in the T_2_Z_1_ condition. Black lines and square markers are relative to the excluded subject group.

To control whether the inter-individual differences reported above were specific of the selected participants (6/14), data from all participants were also analyzed. Figure 8 shows the wrist trajectories in the sagittal plane, averaged across trials, and the scatter plot of the x and z velocities at impact in the T_2_Z_1_ condition for all the 14 subjects enrolled in the experiment. Both caught and touched trials were included, and no exclusion criterion on ball arrival height was applied. Results from the wrist path in the sagittal plane showed that the excluded subjects did not belong to a particular performance group: again, different subjects behaved differently (Figure 8, *left*). The LMM and LM were used to model the data set and to test for the effects of both the different flight times and ball arrival heights (fixed effects) and the different subjects (random effect) on the x-z velocity components at impact. Inter-individual variability in the velocity at impact in the T_2_Z_1_ condition (Figure 8, *right*) was indicated by the strong reduction of the AIC value of the LMM with respect to the LM (Table 1). Notably, there were no clear differences in arm kinematic between touched and caught trials for the selected subjects group. For example, the inclusion of the touched trials did not change the mean velocity at impact (compare Fig. 3 and 8). Although not shown in Figure 6, similar considerations were valid also for the other T-Z flight conditions.

## Discussion

Most previous studies of interceptive movements have focused on the identification of general tendencies in motor control, valid across all tested subjects. Here, instead, we have investigated whether different control strategies are possible and equally successful when subjects can choose *where* and *when* to intercept a moving target. In particular, inter-individual variability in upper limb motion has been characterized by means of several kinematic parameters during a one-handed, unconstrained catching task. Our results showed that different subjects may use different solutions to catch a flying ball successfully.

### Summary of results

Large inter-individual variability was observed in several kinematic features, such as wrist trajectory, velocity profile, timing and spatial distribution of the impact point, body postures and submovement decomposition structures (Figures 2,3,4,5,6,7). Overall we characterized different and idiosyncratic catching movements across participants. S_2_ and S_5_ tended to intercept the target forward away from the trunk, although with slightly different modalities: S_2_ displaced both the body and the arm toward the incoming target and approached the ball with a typical hook-like wrist path, while S_5_ remained still with the body. S_4_ caught the ball backward, close to the trunk, gaining extra time for movement execution and adjustments. Finally, S_1_, S_3_, and S_6_ tended to catch the ball in the middle of the reachable portion of its trajectory while moving toward it either exploiting horizontal trunk motion (S_6_), or with a hook-like trajectory combined with downward trunk motion (S_1_), or moving only the arm (S_3_). These idiosyncratic behaviors were observed in participants with overall comparable performance levels and were maintained across trials, different ball flight duration conditions, and experimental sessions carried out at one year distance (Figure 2). Hence, they were not the result of practice with our task or tentative motor control solutions being explored during performance optimization. Rather, they appeared to reflect a consolidated subject-specific motor strategy. However, in accordance with many previous studies [6], [9], [13], [15], [16], [37], [40], [41], [42], [43], [44], [45], [46], [47], [48], all subjects similarly modulated motion features as a function of target motion characteristics (Table 1). Also in line with previous research [27], [39], the analysis of submovements provided additional evidence that subject-specific end-point kinematic features corresponded to differences in the underlying movement structures which were described by three submovement composition types differing from each other in the number of components and their modulation (Figures 6,7).

### Implication for interceptive control strategies

Whether we consider the catching action to be driven by a prediction of the time and place of the interception [9], [49], [50], [51], by on-line movement control based on continuous monitoring of target motion [10], [40], [52], [53], or by optimal feedback control [54], most of the models developed to describe interception mechanisms do not explicitly address inter-individual variability. However, individual differences in motor behavior might be explained by allowing specific model parameters to vary across subjects. For instance, if interceptive actions are visually guided by on-line control of hand speed according to the perceived target kinematics, as assumed by the required velocity model (RV) [40] or the required velocity integration model (RVITE) [55], a large range of different hand motions may be generated by changing the parameters of the activation function α(t) [56]. Similarly, if the “right time and right place” of the impact is estimated by a combination of interiorized knowledge of the physical environment (i.e. effects of gravity field, mass and size of the ball) with on-line visual information on target motion [51], [57], [58], [59], subject-specific sensitivity to different inputs to the estimation process (e.g., [20]) might lead to different estimates and, consequently, different interceptive motions. Finally, if interceptive movements are controlled through optimal feedback control [54], inter-individual variability in motor performance may arise due to subject-specific parameters in the task-specific cost functions. In this context, Liu and coworkers [60] recently applied optimization techniques to capture and synthesize different movement styles of natural human locomotion. The method assumes that differences in biomechanics such as mechanical properties of tendons and ligaments, relative preferred muscles activation and emotional state (i.e. happy or unhappy mood) are responsible for different locomotor styles. It has also been shown that individual differences in minimizing energy changes of the body segments could also play a role in the manifestation of subject-specific walking characteristics [61]. To date, however the role of other factors (such as past experiences, cognitive processes, proprioceptive features and attention), which may underlie possible differences in the cost function, remains to be explored.

### Possible sources of inter-individual differences in control parameters

Variability has long been recognized as characteristic of human movements [62]. In terms of movement kinematics, when goal-equivalent solutions exist for a given task, e.g. different endpoint trajectories for the same reaching target or different joint angles for the same endpoint spatial position, variability has often been observed to be larger along task irrelevant degrees of freedom [63], [64], suggesting that control is exerted to overcome intrinsic sensorimotor noise mainly when it affects task performance [65]. However, most previous motor control studies have focused on the variability observed across repetitions of the same movement or posture, i.e. on trial-to-trial variability, rather than on variability in the performance of the same task across individuals. In the context of inter-individual variability, the existence of different goal-equivalent solutions, such as the different interception points along the path of the ball observed in the present study, might also be the result of processes involved in stabilizing or improving task performance but, in contrast to trial-to-trial variability, overcoming noise in the acquisition of the appropriate parameters of the control policies rather than execution noise in sensorimotor control: thus, processes more related to skill acquisition than motor execution. However, the results of the present study did not provide evidence of an underlying slow learning process as participants showed a stable and repeatable behavior of kinematics parameters, even at one year distance. On the other hand, they did show a performance improvement after the very first trial of each block. Hence, the origin of the diversity across subjects observed in our experiment would have to be ascribed to processes occurring at some point in life, perhaps during the initial acquisition of catching or similar skills.

It is well known in sport science that besides personal talent or predisposition to the particular sport discipline, an athlete has to practice in order to enhance his/her performance. The learning process underlying the acquisition of the “right technique” may be directed at the development of a forward model of the task, at various levels of representation, which allows to plan motion in every situation [23]. Moreover, the acquired technique might be retained over the long term and transferred to other activities [66], [67], [68]. For example, even after a long period of physical inactivity, a sport player, not necessarily a professional, will be more efficient or at least will show a better coordination in the particular or a similar task than a naive performer [51]. Sometimes this could be related to the capability of paying attention to some selected visual sources of information, rather than to physical conditions [23]. Thus, it is plausible that the different movement styles observed in our experiments were influenced by subject's sport life history (not intended here as level of expertise). Even if the selected participants of this study were not professional athletes in ball sports, some of them had played sports (volleyball S_5_, rugby S_2_ and S_3_), while the other participants referred not to be usual sports player (S_4_ and S_6_), and no information was available for S_1_. However, catching a flying object is a common task that everybody had likely experienced. Furthermore, only subjects showing similar performance level were compared in the main study. Finally, in line with Bartlett and coworkers [25], the observed inter-subject variability in the present study may be “the result of the individual-specific self organization process; performers find unique solutions to the task, although some of these solutions may be sub-optimal”, but, we would add, adequate. In other words, variability could emerge from the different manner of matching task redundancy and the “intrinsic dynamic” of the performer motor system (i.e. the individuals motor system's coordination patterns) [69]. Similar conclusions were also recently proposed by Ganesh and coworkers [70], who stated that our CNS does not implement a global optimization function, but that instead different sub-optimal solutions are possible in relation to motor memory, as well as error and effort minimization.

In conclusion, different but goal-equivalent motor control solutions are implemented when task constraints are relaxed, as for catching a flying ball in three dimensional space. A single sensorimotor control strategy with subject-invariant parameters seems to be unable to fully explain the range of different kinematic features observed across the subjects of our experiments. Instead, retention of previously acquired forward models of experienced tasks, and the ability in capturing and use salient environmental information sources, together with the internal coordination tendencies of individual motor system might play a role in the manifestation of subject-specific motor behaviors. Visuomotor control theories should then also take into account individual factors and further studies will be required to understand their origin.
